# Safety and efficacy of dexamethasone intravitreal implant for treatment of macular edema secondary to retinal vein occlusion in Chinese patients: randomized, sham-controlled, multicenter study

**DOI:** 10.1007/s00417-017-3831-6

**Published:** 2017-11-08

**Authors:** Xiaoxin Li, Ningli Wang, Xiaoling Liang, Gezhi Xu, Xiao-Yan Li, Jenny Jiao, Jean Lou, Yehia Hashad, China Ozurdex in RVO Study Group

**Affiliations:** 1 People’s Eye Center, Peking University People’s Hospital, Xizhimen South Street 11, Beijing, 100044 China; 2 Key Laboratory of Vision Loss and Restoration, Ministry of Education, Beijing, China; 3 Beijing Tongren Eye Center, Beijing Institute of Ophthalmology, Beijing Tongren Hospital, Capital Medical University, Beijing, China; 4 State Key Laboratory of Ophthalmology, Zhongshan Ophthalmic Center, Guangzhou, China; 5 Department of Ophthalmology, Eye and ENT Hospital of Fudan University, Shanghai, China; 6 Allergan plc, Irvine, CA USA

**Keywords:** Corticosteroid, Drug delivery system, Intravitreal injection, Macular edema, Randomized controlled trial, Retinal vein occlusion

## Abstract

**Purpose:**

The purpose of this study was to evaluate the safety and efficacy of dexamethasone intravitreal implant 0.7 mg (DEX) for treatment of macular edema associated with retinal vein occlusion (RVO).

**Methods:**

This study was a six-month, randomized, double-masked, sham-controlled, multicenter, phase 3 clinical trial with a 2-month open-label study extension. Patients with branch or central RVO received DEX (*n* = 129) or sham procedure (*n* = 130) in the study eye at baseline; all patients who met re-treatment criteria received DEX at month 6. Efficacy measures included Early Treatment Diabetic Retinopathy Study (ETDRS), best-corrected visual acuity (BCVA), and central retinal thickness (CRT) on optical coherence tomography.

**Results:**

Time to ≥15-letter BCVA improvement from baseline during the first 6 months (primary endpoint) was earlier with DEX than sham (*p* < 0.001). At month 2 (peak effect), the percentage of patients with ≥15-letter BCVA improvement from baseline was DEX: 35%, sham: 12%; mean BCVA change from baseline was DEX: +10.6 letters, sham: +1.7 letters; and mean CRT change from baseline was DEX: −407 μm, sham: −62 μm (all *p* < 0.001). Outcomes were better with DEX than sham in both branch and central RVO. The most common treatment-emergent adverse event was increased intraocular pressure (IOP). Increases in IOP generally were controlled with topical medication. Mean IOP normalized by month 4, and no patient required incisional glaucoma surgery.

**Conclusions:**

DEX had a favorable safety profile and provided clinically significant benefit in a Chinese patient population with RVO. Visual and anatomic outcomes were improved with DEX relative to sham for 3–4 months after a single implant.

## Introduction

Retinal vein occlusion (RVO) is a common vision-threatening disease of the retina. In the population-based Beijing Eye Study, the estimated prevalence of RVO in adults 40 years of age and older in the Greater Beijing region was 0.7% and increased with age [1]. The two main types of the disease are branch RVO (BRVO) and central RVO (CRVO). Macular edema (ME) is a major cause of vision loss in both BRVO and CRVO [2].

Treatment options for ME associated with RVO include intravitreal anti-vascular endothelial growth factor (anti-VEGF) therapy, intravitreal corticosteroid therapy, and in BRVO, grid laser photocoagulation. Intravitreal anti-VEGF therapy often is effective in treatment of RVO-related ME, but frequent intravitreal injections are required, and not all patients respond to anti-VEGF therapy [3]. Intravitreal corticosteroids are a logical choice for treatment in RVO because inflammation has an important role in the pathogenesis of RVO and RVO-associated ME [4], and corticosteroids have broad antiinflammatory activity. Steroid-related increases in intraocular pressure (IOP) and cataract development are common side effects of intravitreal corticosteroid treatment [5, 6].

Dexamethasone intravitreal implant (DEX; Ozurdex, Allergan plc, Dublin, Ireland) is a biodegradable, sustained-release intravitreal implant containing the potent corticosteroid dexamethasone in the NOVADUR® solid polymer drug delivery system [7]. The implant is delivered into the vitreous through the pars plana using a single-use, 22-gauge applicator [8]. Studies in nonhuman primates have shown that vitreous concentrations of dexamethasone are high for 2 months after DEX administration, then decline but remain measurable for up to 6 months [9]. In the global GENEVA clinical study, DEX 0.7 mg and 0.35 mg were compared with sham procedure in patients with ME secondary to BRVO or CRVO [6, 10]. DEX 0.7 mg and 0.35 mg were superior to sham procedure in reducing central retinal thickness (CRT) and improving best-corrected visual acuity (BCVA) through 3 months after administration of a single implant [6, 10]. The onset of treatment benefit was rapid; patients treated with DEX 0.7 mg were significantly more likely than patients treated with sham procedure to have 3-line improvement in BCVA at 1 week after treatment [11].

On the strength of the GENEVA study results, DEX 0.7 mg was approved in the United States and Europe for treatment of patients with RVO-associated ME. However, only 38 patients with race identified as Asian were treated with DEX 0.7 mg in the GENEVA study [10], and the safety and efficacy of DEX 0.7 mg for treatment of RVO-associated ME has not been well studied in Asian populations. As the prevalence of primary open-angle glaucoma and primary angle-closure glaucoma in adults age 40 years and older in mainland China has been estimated as 0.7% and 1.4%, respectively [12], it may be particularly important to evaluate the incidence and sequelae of steroid-related IOP increases and confirm the safety of DEX treatment in Chinese patients. The objective of the present study was to evaluate the safety and efficacy of DEX 0.7 mg compared with sham procedure in Chinese patients with ME due to BRVO or CRVO.

## Methods

This randomized, double-masked, sham-controlled, multicenter (13 sites), 6-month phase 3 study with a 2-month open-label study extension evaluated the treatment effects of DEX 0.7 mg in patients with ME secondary to RVO. Safety and efficacy of a single DEX 0.7 mg compared with sham procedure were evaluated in the randomized phase of the study. The safety of DEX 0.7 mg treatment was further evaluated in the open-label study extension, in which all patients eligible for treatment received DEX 0.7 mg. The study was conducted in China between September 2012 and May 2014 in compliance with China Good Clinical Practice regulations and guidelines. At the time of the study initiation, no anti-VEGF treatment for RVO-associated ME had been approved in China. The study was designed to be similar to the GENEVA global registration study to allow determination of comparable DEX 0.7 mg treatment effects in a Chinese patient population. The study protocol was approved by an Independent Ethics Committee at each site, and all patients provided written informed consent. The study is registered with the identifier NCT01660802 at www.ClinicalTrials.gov.

### Patient eligibility

Patient eligibility for the study was evaluated at a screening visit (day −14 to −1) and at baseline (day 1). Adults at least 18 years of age with fovea-involved ME in the study eye (defined as macular thickening involving the center of the macula on optical coherence tomography [OCT]), which was due to branch retinal vein occlusion (BRVO) or central retinal vein occlusion (CRVO), were potentially eligible for the study. The ME in the study eye was required to be associated with a decrease in visual acuity, and the duration of ME prior to screening was required to be 6 weeks to 12 months for BRVO and 6 weeks to 9 months for CRVO. The BRVO or CRVO in the study eye was required to be non-ischemic based on the investigator’s evaluation of fluorescein angiography (FA). Best-corrected visual acuity (BCVA) measured with the Early Treatment Diabetic Retinopathy Study (ETDRS) method [13] was required to be ≥34 and ≤68 letters (20/200 and 20/50 Snellen equivalent) in the study eye at screening. CRT in the 1-mm central macular subfield was required to be ≥320 μm on OCT using the Spectralis machine (Heidelberg Engineering, Heidelberg, Germany) or ≥300 μm using the Cirrus machine (Zeiss, Oberkochen, Germany) in the study eye at screening.

Key exclusion criteria for study eyes included ischemic RVO (defined as more than ten disc areas of retinal capillary non-perfusion involving the center of the macula on FA); history of glaucoma; intravitreal steroid or other intravitreal drug use within 3 months before baseline; intraocular surgery or laser therapy within 3 months before baseline; and presence of any ocular condition that, in the opinion of the investigator, would prevent a 15-letter improvement in BCVA (such as severe macular ischemia, epiretinal membrane, or foveal atrophy) or might affect ME or BCVA during the study. Eyes with media opacity at the screening visit that precluded clinical and photographic evaluation (including, but not limited to, preretinal or vitreous hemorrhage and lens opacity) and eyes with dense macular hemorrhage with no red reflex present were also excluded. Other key exclusion criteria included history of pars plana vitrectomy in the study eye; active bacterial, viral, parasitic, or fungal infection in either eye; history of IOP elevation in response to steroid treatment in either eye; use of oral, intravenous, intramuscular, epidural, rectal, or extensive dermal steroid within 1 month before baseline; use of immunosuppressant, immunomodulator, antimetabolite, and/or alkylating agent within 3 months before baseline; use of topical ophthalmic corticosteroid or prescribed Chinese herbal medication within 2 weeks before baseline; BCVA score of <34 letters in the non-study eye; improvement in BCVA of >10 letters between screening and baseline; and any condition that in the investigator’s opinion might confound the study results.

### Study treatment

At baseline (day 1), eligible patients were enrolled and randomized in a 1:1 ratio to treatment with DEX 0.7 mg or sham procedure in the study eye. The randomization at each site was stratified by study eye diagnosis (BRVO or CRVO). Patients received study treatment following baseline evaluations. For patients assigned to DEX, DEX 0.7 mg was administered by intravitreal injection using a single-use applicator system [8]. For patients assigned to the sham procedure, a needleless applicator was pressed against the conjunctiva.

Eligibility for a second study treatment was evaluated at month 6. Re-treatment was allowed if BCVA in the study eye was <84 letters (~20/20 or worse Snellen equivalent), there was evidence of residual edema (CRT >250 μm, intraretinal cysts, or regions of increased retinal thickening within or outside the center subfield), and in the investigator’s opinion, the procedure would not put the patient at significant risk. All patients from both the DEX and sham groups who were re-treated at month 6 received DEX 0.7 mg in the study eye.

### Rescue treatment

Rescue treatment with laser photocoagulation was permitted if it had been ≥3 months since administration of the study treatment and BCVA had dropped by ≥10 letters from day 1, due to worsening of ME associated with RVO, at two consecutive visits at least 4 weeks apart. Under any circumstances, rescue laser treatment could be given if the investigator considered it to be in the best interest of the patient. Patients who received rescue laser treatment remained in the study and could receive open-label treatment with DEX at month 6.

### Visits and assessments

Patients were seen at monthly study visits from month 1 through month 8. Additional safety assessments were made by the treating investigator 1 day after treatment and re-treatment. Efficacy evaluations at the monthly visits included BCVA measured using the ETDRS method and OCT. Fluorescein leakage on FA was assessed at screening and month 6. Masked readers of the FA at the central reading center determined the presence and area of neovascularization from gradable images. Key safety evaluations at all visits included biomicroscopy/ophthalmology, IOP measured with a Goldmann applanation tonometer, and treatment-emergent adverse events (TEAEs). Investigators graded the presence and severity of nuclear, cortical, and posterior subcapsular lens opacities on biomicroscopy using the Age-Related Eye Disease Study Clinical Lens Grading System [14], by comparing the biomicroscopic findings with standard photographs. A TEAE was defined as an adverse event that had onset or an increase in severity after baseline, or any serious adverse event. All patients, investigators who followed the patients at the monthly visits, study personnel who collected efficacy data, and readers at the central reading center (Doheny Image Reading Center, Los Angeles, California, USA) who evaluated the OCT images were masked to the treatment assignment.

### Outcomes measures

The primary efficacy endpoint was the time to achievement of ≥15-letter improvement in BCVA from baseline in the study eye during the first 6 months. Key secondary endpoints at each visit through month 6 included study eye mean BCVA change from baseline, the percentage of patients with ≥15-letter improvement in BCVA from baseline in the study eye, and study eye mean CRT change from baseline on OCT. The average change in BCVA from baseline in the study eye during the first 6 months was also evaluated using an area-under-the-curve (AUC) approach. Subgroup analysis by RVO diagnosis (BRVO or CRVO) was performed for key endpoints.

### Data analysis and statistical methods

The modified intent-to-treat (mITT) population consisting of all randomized and treated patients was used for the primary efficacy analyses. Supportive analysis of the primary endpoint used the per-protocol population of all randomized and treated patients with no major protocol violations. Safety parameters were evaluated in the safety population of all treated patients, based on the actual treatment received.

Statistical analysis used SAS version 9.3 (SAS Inc., Cary, North Carolina, USA) and a 2-sided alpha level of 0.05. Data after rescue treatment were not used for any efficacy analysis; all efficacy data after rescue treatment were set to missing, and missing values were imputed with the last-observation-carried-forward method.

Kaplan-Meier survival analysis was used to analyze time to first ≥15-letter improvement in BCVA from baseline during the first 6 months. In the analysis, patients who failed to respond by the month 6 visit were censored at the month 6 visit or on the date of the last visual acuity measurement, if discontinued from the study prior to the month 6 visit. Patients who received rescue treatment before achieving ≥15-letter BCVA improvement were censored at the time of the first rescue treatment. Log-rank tests were used to compare cumulative response rates between groups.

Average BCVA over 6 months was calculated with the AUC approach; the AUC was estimated using the trapezoidal method based on observed data and divided by the number of study days at the last BCVA measurement. Average BCVA was compared between groups using a two-way analysis of variance (ANOVA) with treatment group and RVO diagnosis (BRVO or CRVO) as fixed effects. All other secondary analyses of BCVA and OCT data used ANOVA for continuous variables and the Mantel-Haenszel test for categorical variables with stratification for RVO diagnosis. TEAEs were coded using MedDRA version 17.0 preferred terms, and the overall incidence of any TEAE related to cataract (cataract, cataract diabetic, cataract nuclear, cataract subcapsular, cortical cataract, or lenticular opacities) was evaluated.

The planned sample size of 130 patients in each treatment group provided 85% power to detect a difference between groups in the time to achieve ≥15-letter improvement from baseline in BCVA within the first 6 months, assuming a cumulative response rate of 22.5% for the sham group and a constant hazard ratio of 2 for DEX versus sham.

## Results

A total of 328 patients were screened for inclusion in the study; 262 of those patients were enrolled and randomized to treatment with DEX or sham. Three patients who were enrolled and randomized (one DEX, two sham) were excluded from the mITT population, because they were not treated. Baseline characteristics of patients in the mITT population (*n* = 259) are listed in Table 1. All patients were Asian. By chance, the percentage of male patients was larger (53.5% vs 41.5%) in the DEX group than in the sham group. However, study eye disease characteristics were similar between treatment groups. Approximately half the study eyes were diagnosed with BRVO and half with CRVO, and almost all study eyes (255/259, 98.5%) were phakic.

**Table 1:** Baseline characteristics of patients and study eyes (mITT population)

Characteristic	DEX (*n* = 129)	Sham (*n* = 130)	*p* value
Mean age (SD), years	54.6 (9.8)	53.0 (12.0)	0.016
Range	25–78	19–77	
Gender, n (%)			0.054
Male	69 (53.5)	54 (41.5)	
Female	60 (46.5)	76 (58.5)	
Race, n (%)			NA
Asian	129 (100)	130 (100)	
Diagnosis, n (%)			0.852
BRVO	63 (48.8)	65 (50.0)	
CRVO	66 (51.2)	65 (50.0)	
Mean duration of ME in eyes with BRVO (SD), days	125 (86)	113 (73)	0.571
Duration, n (%)			
≤90 days	32 (50.8)	36 (55.4)	
>90 days	31 (49.2)	29 (44.6)	
Mean duration of ME in eyes with CRVO (SD), days	124 (59)	130 (67)	0.742
Duration, n (%)			
≤90 days	23 (34.8)	25 (38.5)	
>90 days	43 (65.2)	40 (61.5)	
Previous ocular procedure for RVO, n (%)^a^	15 (11.6)	12 (9.2)	0.528
Lens status, n (%)			0.370
Phakic	126 (97.7)	129 (99.2)	
Pseudophakic	3 (2.3)	1 (0.8)	
Mean IOP (SD), mm Hg	14.9 (2.9)	15.0 (2.7)	0.770
Mean BCVA (SD), letters	52.6 (10.8)	53.1 (10.5)	0.726
Mean CRT (SD), μm	683 (242)	643 (213)	0.149

*BCVA* best-corrected visual acuity, *BRVO* branch retinal vein occlusion, *CRT* central retinal thickness, *CRVO* central retinal vein occlusion, *DEX* dexamethasone intravitreal implant, *IOP* intraocular pressure, *ME* macular edema, *mITT* modified intent-to-treat, *NA* not available, *RVO* retinal vein occlusion, *SD* standard deviation

^a^Previous ocular procedures for RVO included intravitreal injections of anti-vascular endothelial growth factor, intravitreal or intraocular injections of corticosteroid, and laser

**Fig. 1:**
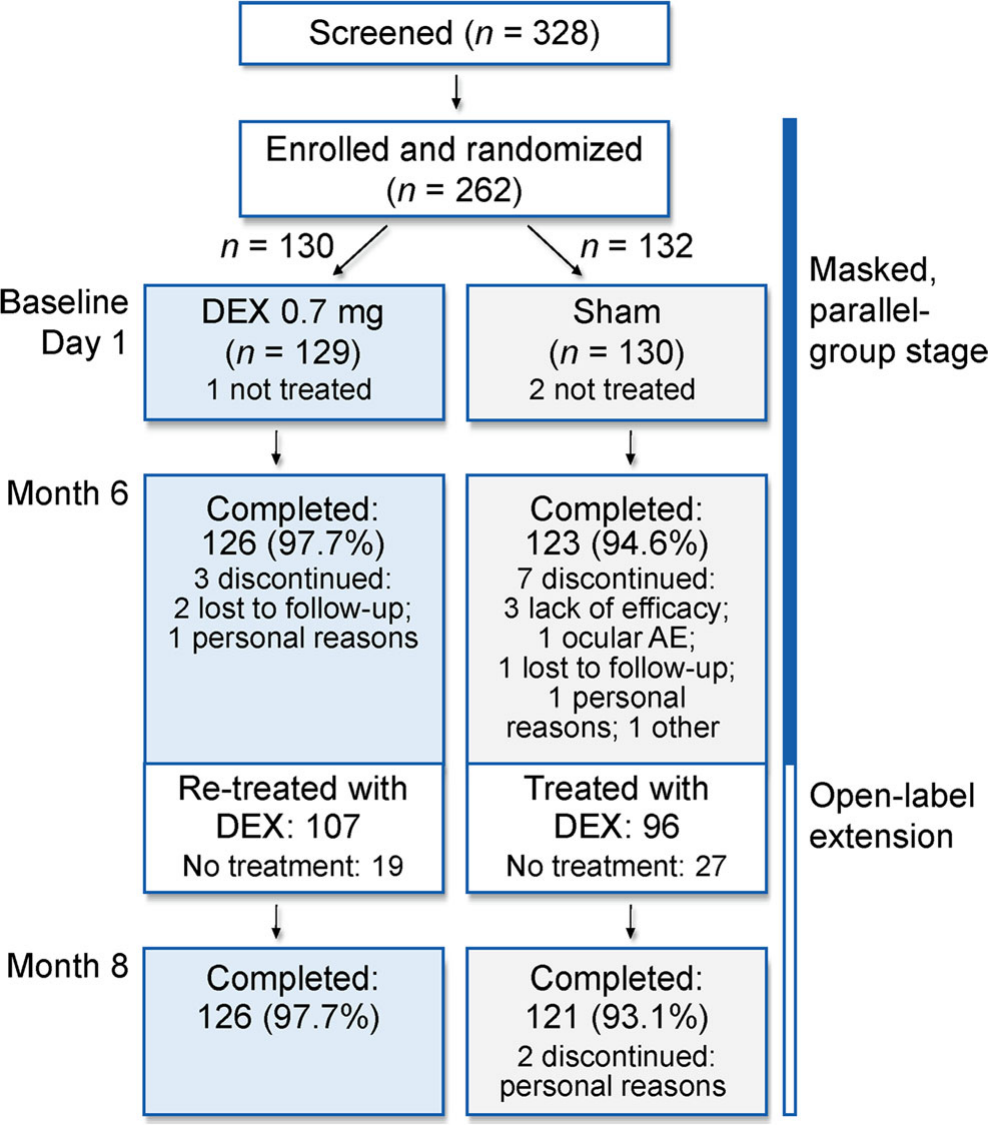
Patient flow through the study (mITT population). *AE* adverse event, *DEX* dexamethasone intravitreal implant, *mITT* modified intent-to-treat

Fig. 1 shows patient flow through the study for the mITT population. The 6-month, double-masked stage of the study was completed by 97.7% (126/129) of patients in the DEX group and 94.6% (123/130) of patients in the sham group. The only discontinuation due to an adverse event was in the sham group; one patient in the sham group discontinued due to cystoid macular edema.

At month 6, 203 patients (107 in the DEX group [53 BRVO, 54 CRVO] and 96 in the sham group [41 BRVO, 55 CRVO]) received open-label DEX treatment. Forty-six patients (30 BRVO, 16 CRVO) of the 249 patients who remained in the study were not treated at month 6. In the majority of these cases (25/46, 54.3%), treatment was not needed because the CRT in the study eye was ≤250 μm. All patients (100%, 107/107) in the DEX group who received a second DEX treatment and 99% (95/96) of patients in the sham group who received initial treatment with DEX at month 6 completed the 2-month open-label study extension.

Rescue retinal laser photocoagulation was administered during the study to 10.9% (14/129) of patients in the DEX group (seven with BRVO; seven with CRVO) and 8.5% (11/130) of patients in the sham group (nine with BRVO; two with CRVO). For the majority of these patients (10/14, 71.4% in the DEX group and 6/11, 54.5% in the sham group), the first retinal laser treatment was at month 6.

### Efficacy outcomes

**Fig. 2:**
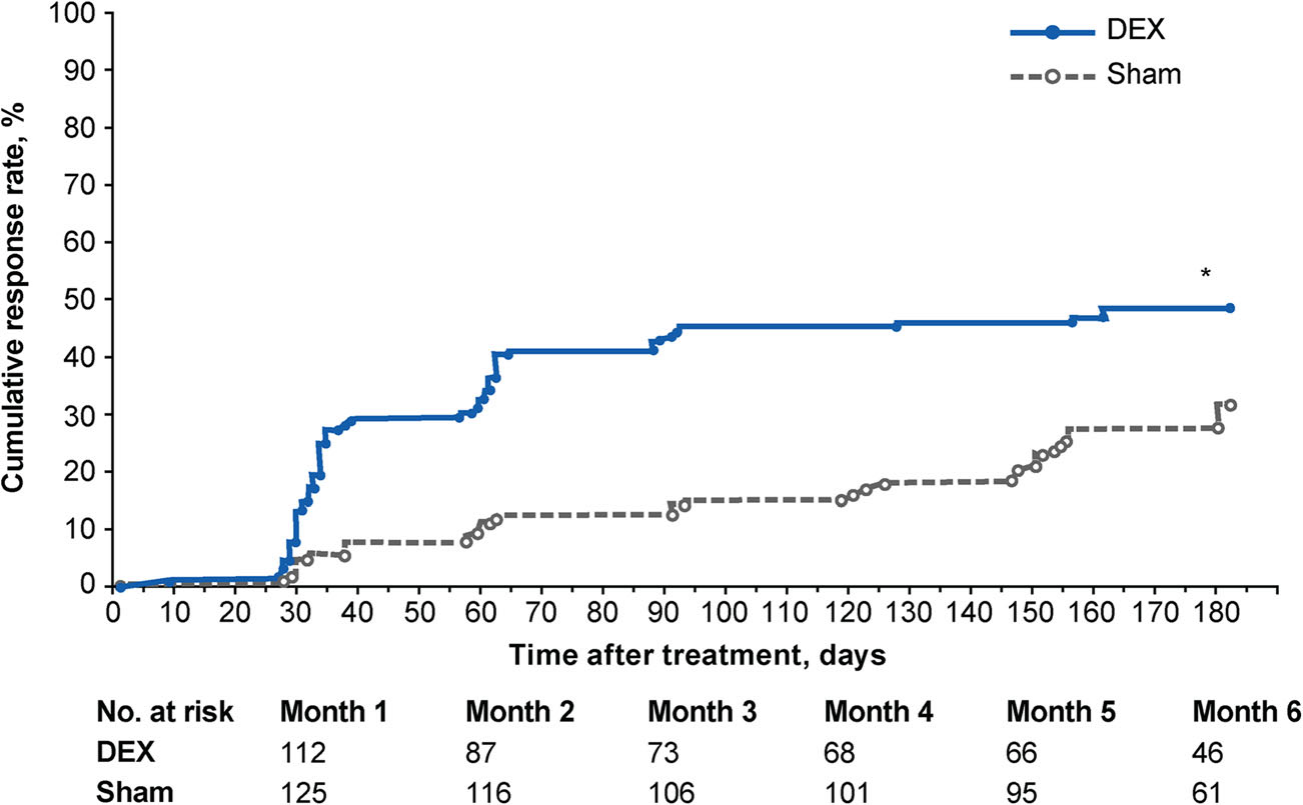
Kaplan-Meier analysis of the time to ≥15 ETDRS letters improvement in best-corrected visual acuity from baseline (mITT population). *p < 0.001 versus sham (log rank test comparing cumulative response rate curves across time). *DEX* dexamethasone intravitreal implant, *ETDRS* Early Treatment Diabetic Retinopathy Study, *mITT* modified intent-to-treat

DEX provided rapid improvement in BCVA. Survival analysis showed significantly earlier ≥15-letter improvement in BCVA in the DEX group compared with the sham group (*p* < 0.001) (Fig. 2). Separation of the cumulative response rate curves for the DEX and sham groups was evident by the first efficacy visit (month 1) and maintained through month 6. The significant overall difference in response rates between treatment groups (*p* < 0.001) was confirmed in the per-protocol population and in a Cox regression model that adjusted for baseline RVO diagnosis, age, and sex. The estimated hazard ratio (95% confidence interval) from the model was 2.4 (1.6, 3.7), indicating a 2.4-fold higher rate of achieving ≥15 letters gain with DEX compared with sham.

**Fig. 3:**
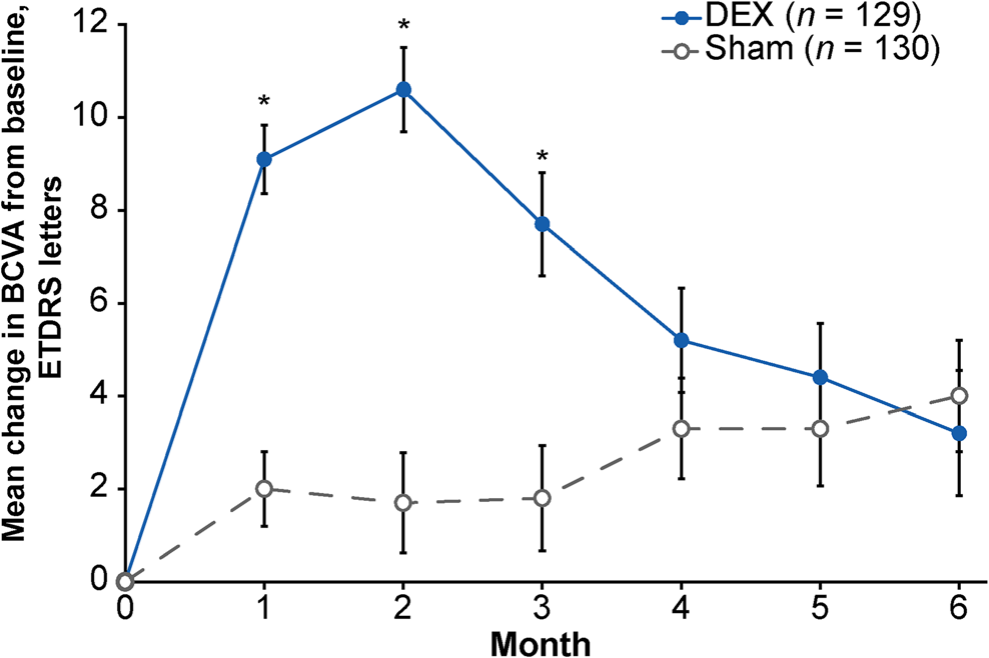
Mean change in BCVA from baseline (mITT population). Error bars represent the standard error of the mean. *p < 0.001 versus sham. *BCVA* best-corrected visual acuity, *DEX* dexamethasone intravitreal implant, *ETDRS* Early Treatment Diabetic Retinopathy Study, *mITT* modified intent-to-treat

**Fig. 4:**
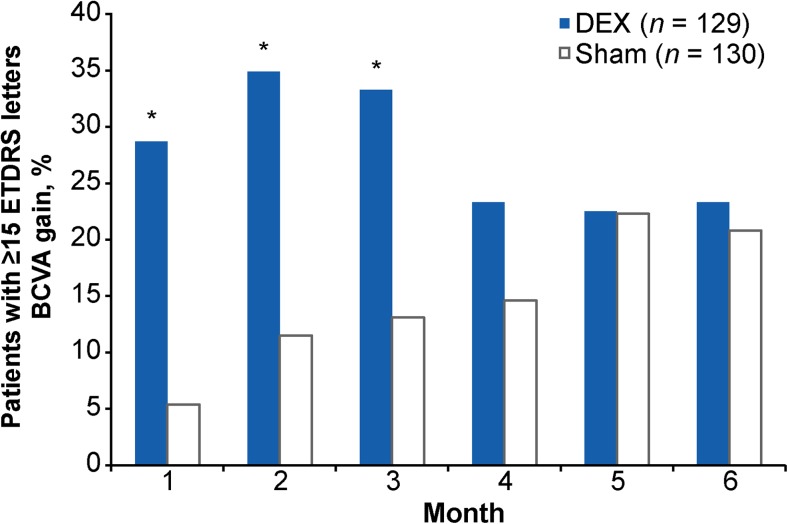
Percentage of patients with ≥15-letter gain in BCVA from baseline (mITT population). *p < 0.001 versus sham. *BCVA* best-corrected visual acuity, *DEX* dexamethasone intravitreal implant, *ETDRS* Early Treatment Diabetic Retinopathy Study, *mITT* modified intent-to-treat

Mean change in BCVA from baseline (Fig. 3) and the percentage of patients with ≥15-letter improvement in BCVA from baseline (Fig. 4) were significantly larger in the DEX group compared with the sham group at months 1, 2, and 3 (*p* < 0.001). At month 2 (peak effect), mean (standard deviation, SD) BCVA change from baseline was +10.6 (10.4) letters with DEX versus +1.7 (12.3) letters with sham (*p* < 0.001), and the percentage of patients with ≥15-letter BCVA improvement from baseline was 34.9% with DEX versus 11.5% with sham (*p* < 0.001). The mean (SD) average change in BCVA from baseline over 6 months (AUC approach) was 6.7 (9.0) letters with DEX compared with 2.5 (10.0) letters with sham (*p* < 0.001).

**Fig. 5:**
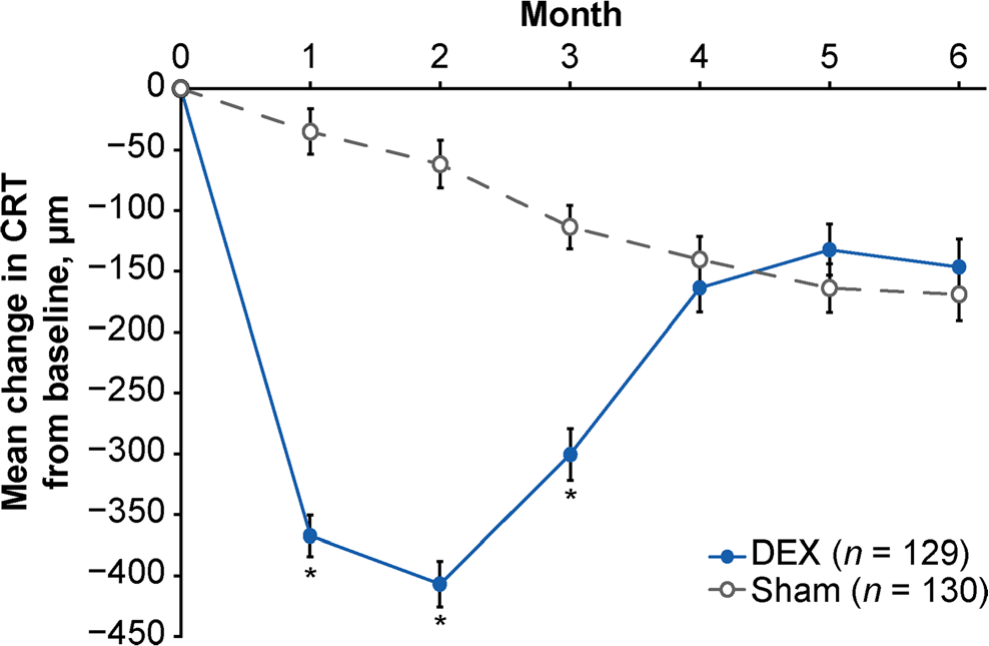
Mean change in CRT from baseline (mITT population). Error bars represent the standard error of the mean. *p < 0.001 versus sham. *CRT* central retinal thickness, *DEX* dexamethasone intravitreal implant, *mITT* modified intent-to-treat

DEX also demonstrated superiority to sham in anatomic outcomes. Mean CRT reduction from baseline was significantly larger in the DEX group compared with the sham group at months 1, 2, and 3 (*p* < 0.001, Fig. 5). At month 2 (peak effect), mean (SD) CRT change from baseline was −407 (212) μm with DEX versus −62 (224) μm with sham (*p* < 0.001).

**Fig. 6:**
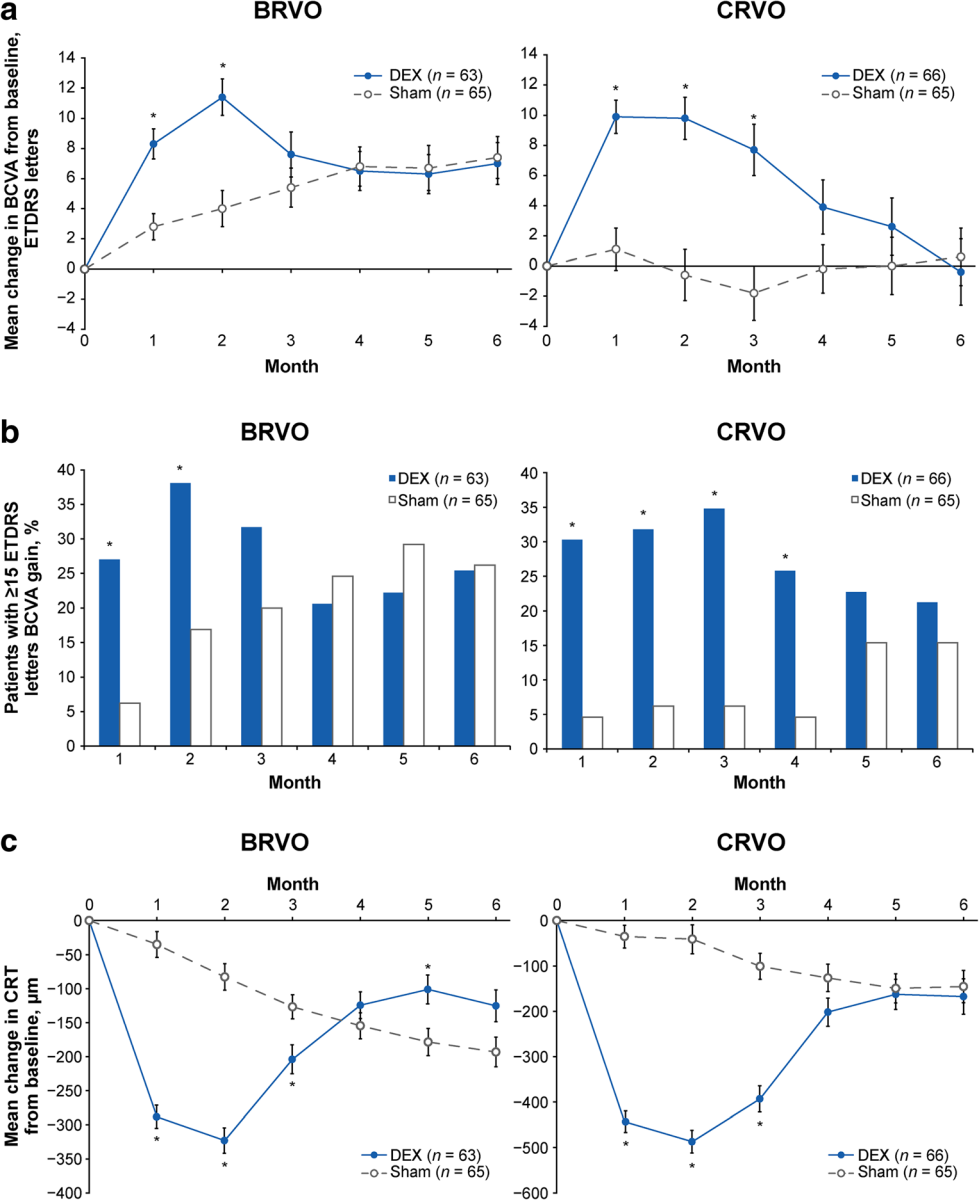
Key efficacy parameters in BRVO and CRVO subgroups (mITT population). **a** Mean change in BCVA from baseline. **b** Percentage of patients with ≥15 letters gain in BCVA from baseline. **c** Mean change in CRT from baseline. *p ≤ 0.028 versus sham. *BCVA* best-corrected visual acuity, *BRVO* branch retinal vein occlusion, *CRT* central retinal thickness, *CRVO* central retinal vein occlusion, *DEX* dexamethasone intravitreal implant, *ETDRS* Early Treatment Diabetic Retinopathy Study, *mITT* modified intent-to-treat

Subgroup analysis based on RVO diagnosis showed that DEX improved BCVA and CRT in both BRVO and CRVO during the first 3 months after treatment (Fig. 6). At month 2, mean (SD) change in BCVA from baseline was +11.4 (9.6) letters in DEX-treated patients with BRVO, +4.0 (10.0) letters in sham-treated patients with BRVO, +9.8 (11.0) letters in DEX-treated patients with CRVO, and −0.6 (13.9) letters in sham-treated patients with CRVO. Mean (SD) change in CRT from baseline at month 2 was −323 (189) μm in DEX-treated patients with BRVO, −83 (187) μm in sham-treated patients with BRVO, −487 (203) μm in DEX-treated patients with CRVO, and −41 (256) μm in sham-treated patients with CRVO. Outcomes were significantly better with DEX compared with sham for 2–3 months in patients with BRVO and for 3–4 months in patients with CRVO (Fig. 6). In both BRVO and CRVO, BCVA outcomes were more favorable in patients with a baseline duration of ME of ≤90 days compared with patients with a baseline duration of ME of >90 days.

At screening on FA, the mean ± SD area of neovascularization in the disc was 5.26 ± 5.96 mm^2^ in the DEX group and 4.13 ± 3.72 mm^2^ in the sham group. At month 6, the mean ± SD reduction from screening was 3.91 ± 7.30 mm^2^ in the DEX group and 2.56 ± 4.58 mm^2^ in the sham group.

### Safety outcomes

TEAEs were reported during the first 6 months of the study in 53.5% (69/129) of patients in the DEX group and 31.5% (41/130) of patients in the sham group. The most common TEAEs were increased IOP, conjunctival hemorrhage, and conjunctival hyperemia (Table 2). There were no reports of any systemic treatment-related TEAE. The profile of TEAEs with onset during the open-label study extension for patients treated or re-treated with DEX at month 6 was similar to the profile of TEAEs seen in patients initially treated with DEX at baseline (Table 3). There were no unexpected TEAEs after a second implant. Cataract-related TEAEs were reported in two (1.6%) patients in the DEX group and no patients in the sham group during the first 6 months. During the study extension, cataract-related TEAEs were reported in two of the 107 patients (1.9%) who were re-treated with DEX. No study eye had cataract surgery during the study.

**Table 2:** Treatment-emergent adverse events during the first 6 months (safety population)^a^

Adverse event preferred term, n (%)^b^	DEX (*n* = 129)	Sham (*n* = 130)
IOP increased	38 (29.5)	4 (3.1)
Conjunctival hemorrhage	24 (18.6)	5 (3.8)
Conjunctival hyperemia	17 (13.2)	6 (4.6)
Visual acuity reduced	4 (3.1)	6 (4.6)
Conjunctival edema	4 (3.1)	1 (0.8)
Ocular hypertension	4 (3.1)	0 (0)
Eye pain	3 (2.3)	3 (2.3)
Headache	3 (2.3)	1 (0.8)
Vitreous hemorrhage	0 (0)	3 (2.3)

*DEX* dexamethasone intravitreal implant, *IOP* intraocular pressure

^a^All treatment-emergent adverse events reported in >2% of patients in either treatment group are listed

^b^Adverse events were categorized for analysis using MedDRA version 17.0 preferred terms

**Table 3:** Treatment-emergent ocular adverse events with onset during the 2-month study extension in patients treated with DEX at month 6 (safety population)^a^

Adverse event, n (%)	Initial DEX treatment and month 6 DEX re-treatment (*n* = 107)	Initial sham treatment and month 6 DEX treatment (*n* = 96)
IOP increased	25 (23.4)	22 (22.9)
Conjunctival hemorrhage	10 (9.3)	10 (10.4)
Conjunctival hyperemia	6 (5.6)	5 (5.2)
Ocular hypertension	3 (2.8)	0 (0)

*DEX* dexamethasone intravitreal implant, *IOP* intraocular pressure

^a^All treatment-emergent ocular adverse events with onset after month 6 that were reported in >2% of patients in either treatment group are listed

Serious adverse events were reported in three patients during the first 6 months (atrioventricular block in the DEX group; vitreous hemorrhage and chronic cholecystitis in the sham group). An additional serious adverse event (cerebral infarction) was reported during the study extension in a patient in the sham group who was treated with DEX. None of the serious adverse events were considered to be related to treatment.

**Fig. 7:**
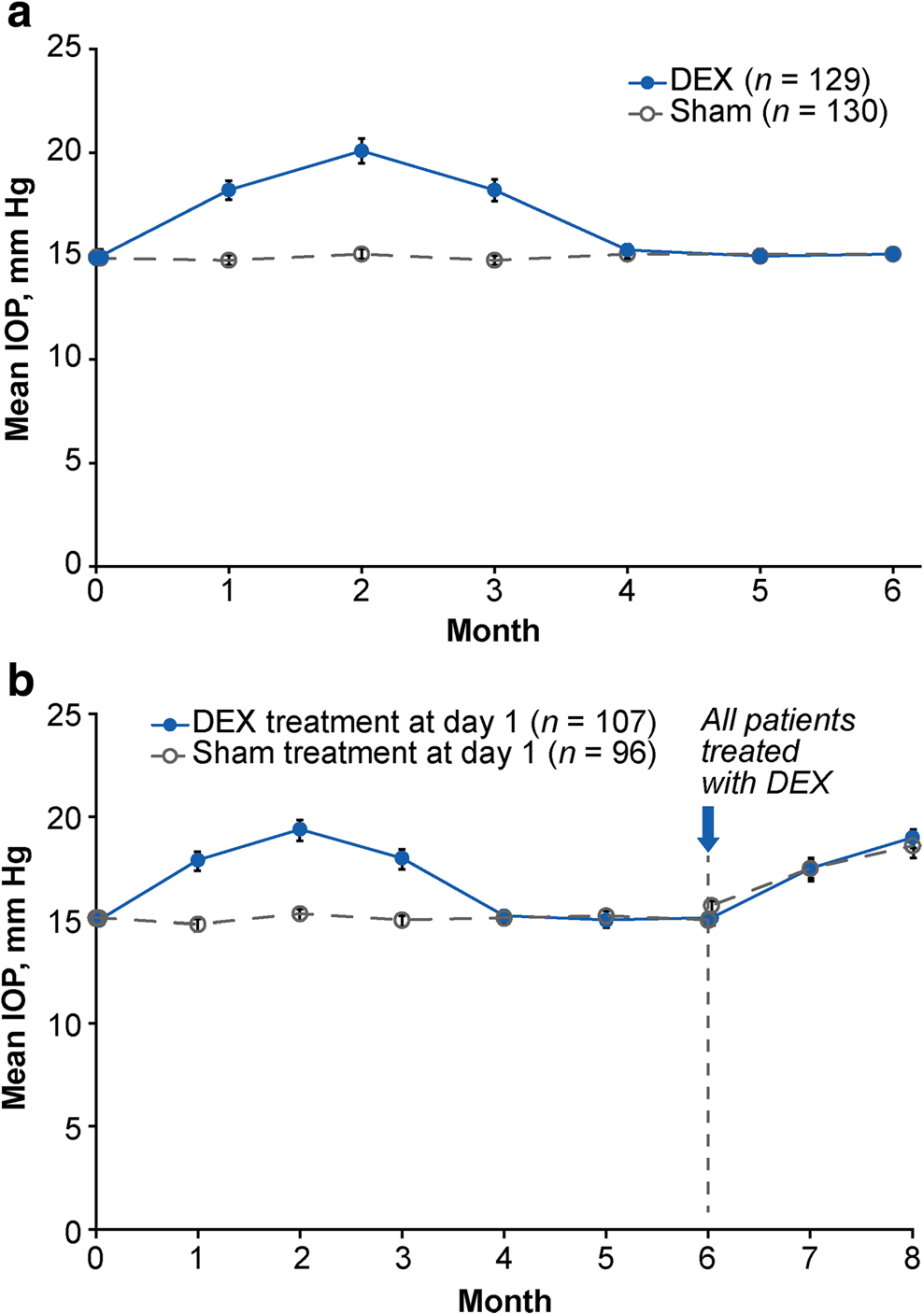
Mean IOP (safety population). **a** Mean IOP after initial treatment in the masked phase of the study. **b** Mean IOP through month 8 in patients who were treated in the open-label study extension. Error bars represent the standard error of the mean. *DEX* dexamethasone intravitreal implant, *IOP* intraocular pressure

During the first 6 months of the study, 27.1% (35/129) of patients in the DEX group compared with 1.5% (2/130) in the sham group had an increase in IOP of at least 10 mm Hg from baseline, 23.3% (30/129) of patients in the DEX group compared with 0.8% (1/130) in the sham group had IOP ≥25 mm Hg, and 6.2% (8/129) of patients in the DEX group compared with 0% (0/130) in the sham group had IOP ≥35 mm Hg. Mean IOP peaked at 2 months and returned to baseline levels by 4 months after DEX treatment (Fig. 7a). For patients treated with DEX at both baseline and month 6, the increase in mean IOP was similar after the initial treatment and re-treatment (Fig. 7b).

Topical IOP-lowering medications were used for IOP management. During the 8-month study, 34.9% (45/129) of patients in the DEX group (who received one or two implants) and 13.8% (18/130) of patients in the sham group (who received no or one implant) used IOP-lowering medication to control IOP elevations. Among patients who used IOP-lowering medications, most (25/45, 55.6% in the DEX group and 15/18, 83.3% in the sham group) used a single medication. One patient in the DEX group underwent laser trabeculoplasty due to increased IOP in the study eye, and one patient in the sham group underwent iridectomy due to glaucoma in the study eye during the initial 6 months of the study. No IOP-lowering procedures were performed during the open-label study extension, and no incisional glaucoma surgeries were performed at any time during the study.

## Discussion

In this study, DEX 0.7 mg provided a clinically significant benefit and was well tolerated in Chinese patients with RVO. DEX provided rapid improvement in BCVA and met the primary endpoint of the study; i.e., patients achieved earlier ≥15-letter BCVA gain with DEX compared with sham procedure. DEX also was superior to sham procedure in secondary efficacy outcome measures. Mean change in BCVA from baseline, the percentage of patients gaining at least 15 letters in BCVA from baseline, and mean change in CRT from baseline were significantly greater with DEX than sham procedure at months 1, 2, and 3. The mean average change in BCVA across 6 months was also significantly greater with DEX than sham procedure. Treatment with DEX improved outcomes in both patients with BRVO and patients with CRVO.

The efficacy and safety of DEX in this Chinese patient population were similar to those demonstrated in the patient population of the global GENEVA study. Yet in the GENEVA study, the mean improvement in BCVA at month 6 was better with DEX 0.7 mg than sham for BRVO patients but not CRVO patients, whereas in the present study, there was no difference between DEX and sham in BCVA improvement at month 6 for either BRVO or CRVO patients, and the benefit of DEX treatment was maintained longer in CRVO than in BRVO, most likely because of the spontaneous improvement of BCVA and CRT in patients with BRVO. It is well established that RVO-associated ME of short duration is most responsive to treatment with DEX [15] or anti-VEGF [16, 17]. However, a difference in disease duration is unlikely to explain this difference in results between GENEVA and the present study, as the mean duration of ME at baseline was similar (approximately 4–5 months) in these studies. Thus, the reasons for the difference in month 6 results in BRVO patients between studies remain unknown, but could involve differences in the patient populations or disease characteristics, or chance.

The design of the GENEVA global study included follow-up assessment of BCVA at days 30, 60, 90, and 180. Peak efficacy was seen at day 60, and by day 180, BCVA outcomes were no longer consistently significantly better in the DEX 0.7 mg group compared with the sham group [10]. Consistent with those results, mean change in CRT from baseline in the GENEVA study was significantly better with DEX 0.7 mg than sham at day 90 but not day 180 [10]. The results suggested a duration of response to DEX treatment somewhere between 90 and 180 days, but the duration could not be narrowly defined because of the absence of follow-up visits between 3 and 6 months. The design of the present study had the advantage of including monthly follow-up, and therefore, this study provided additional information about the time course and duration of DEX treatment effect on BCVA and CRT compared with the GENEVA study. The results showed very similar efficacy outcomes in the DEX and sham groups at months 5 and 6, suggesting a need for a re-treatment interval of 4–5 months for many patients. Consistent with this suggestion, recent studies of the use of DEX 0.7 mg for treatment of RVO in clinical practice have typically reported an average interval between implant injections of less than 6 months [18–20]. For example, in a retrospective study of the use of DEX 0.7 mg in clinical practice in the United States (the SHASTA study), DEX 0.7 mg was effective when used as monotherapy for RVO-associated ME at a re-treatment interval of approximately 5 months [19]. Although a re-treatment interval for DEX of 4–5 months may be needed for optimal efficacy, the frequency of intravitreal injections is still much less with DEX than with anti-VEGF therapy. The reduction in treatment burden associated with intravitreal injections is an advantage of DEX treatment for RVO-associated ME.

The safety profile of DEX for treatment of RVO-associated ME demonstrated in this study was favorable and consistent with previous reports [10, 21]. The TEAEs reported are expected with an intraocular corticosteroid, and no patient treated with DEX discontinued from the study because of a TEAE. The only TEAEs that were more common with DEX than sham were corticosteroid-related increases in IOP and injection-related conjunctival hyperemia and conjunctival hemorrhage. Increases in IOP generally were controlled with topical medications, and no patient in the DEX-treated group required incisional glaucoma surgery. The mean increase in IOP after re-treatment with DEX was similar to that observed after the initial treatment. This is consistent with findings from the 3-year MEAD study of DEX treatment in patients with diabetic ME, which showed no cumulative effect of sequential implants on IOP and no increase in the frequency of IOP elevations after repeat treatment [22]. As expected, mean IOP was elevated over baseline levels at the end of the study, because the study ended 2 months after the open-label DEX treatment. Mean IOP in the MEAD study was shown to peak at 1.5–3 months after treatment and decrease to baseline levels by 6 months after treatment [22].

Cataract TEAEs were reported in only two of 221 patients (0.9%) with phakic study eyes who received one or two DEX injections, and no cataract extractions were performed during the study. The duration of the study, however, was only 8 months, and patients in the initial sham group were not treated with DEX until month 6. Previous studies of DEX use for treatment of RVO have shown that cataract progression and need for cataract surgery are more likely to occur after multiple injections of DEX and a longer duration of treatment [6, 23].

Limitations of this study include the use of a re-treatment interval of 6 months. This allowed us to evaluate the duration of effect of a single implant, but likely resulted in less favorable BCVA and OCT outcomes than if DEX had been administered using a shorter re-treatment interval of 4 or 5 months.

In summary, this study demonstrated that DEX 0.7 mg is effective in improving visual and anatomic outcomes in Chinese patients with BRVO or CRVO. Statistically and clinically significant greater improvement in BCVA and CRT were seen with DEX treatment relative to sham for 3–4 months after a single implant. A re-treatment interval of less than 6 months may be required for optimal outcomes.

## Appendix

### China Ozurdex in RVO Study Group

Nan Chen, MD (Qingdao Eye Hospital, Qingdao, China); Fang Tian Dong, MD (Peking Union Medical College Hospital, Beijing, China); Wei He, MD (Shenyang He Eye Hospital, Shenyang, China); Xiaoxin Li, MD (Peking University People’s Hospital, Beijing, China); Xiaoling Liang, MD (Zhongshan Ophthalmic Center, Sun Yet-Sen University, Guangzhou, China); Xiao Ling Liu, MD (The Affiliated Eye Hospital of Wenzhou Medical College, Wenzhou, China); E Song, MD (The First Hospital of Jilin University, Changchun, China); Luo-sheng Tang, MD (The Second XiangYa Hospital of Central South University, Changsha, China); Ningli Wang, MD (Beijing Tongren Hospital, Beijing, China); Yu Sheng Wang, MD, PhD (The First Affiliated Hospital of the Fourth, Military Medical University, Xi’an, China); Gezhi Xu, MD (The Eye and ENT Hospital, Affiliated of Fudan University, Shanghai, China); Xun Xu, MD (Shanghai First People’s Hospital, Shanghai, China); and Jun Jun Zhang, MD (West China Hospital, Sichuan University, Chengdu, China).
